# Clinical Outcomes of the Induced Membrane Technique for Infected Non-unions and Post-traumatic Bone Defects in a Resource-Limited Setting

**DOI:** 10.7759/cureus.104038

**Published:** 2026-02-21

**Authors:** Sumit Dabhi, Yogi Dhandhiya, Gaurang Patel

**Affiliations:** 1 Department of Orthopaedics, Medical College Baroda and Sir Sayajirao General Hospital, Vadodara, IND

**Keywords:** bone defects, induced-membrane technique, infected non-union, limb-salvage, resource-limited setting

## Abstract

Background

Post-traumatic bone defects and infected non-unions pose significant challenges in orthopedic trauma surgery, particularly in low-resource settings. The induced membrane technique (IMT) offers a promising solution, but evidence from resource-constrained environments remains limited. This study evaluates the efficacy of IMT in such settings.

Methods

A prospective cohort study was conducted from May 2023 to May 2024 at a tertiary care hospital in Vadodara, India. Thirty patients with infected non-unions or bone defects (>3 cm) in long bones were enrolled. The two-stage IMT protocol included radical debridement with antibiotic-loaded polymethylmethacrylate (PMMA) spacer placement, followed by autologous bone grafting. Outcomes were assessed using the Association for the Study and Application of the Method of Ilizarov (ASAMI) scoring system at six weeks, three months, six months, and 12 months postoperatively.

Results

Among 20 analyzed patients (mean age: 38 years; 75% male), 80% achieved excellent or good outcomes. Union rates were higher in younger patients (<40 years; 85% success rate) and smaller defects (<5 cm; 88% success rate). Complications included deformity (10%) and persistent infection (5%). Infection control was successful in 95% of cases. Stratified analysis revealed better outcomes in trauma-related defects (75%) compared to chronic non-unions (25%).

Conclusion

IMT is a viable, cost-effective option for managing complex bone defects in resource-limited settings, with high success rates and low reinfection risks. Younger age, smaller defects, and absence of comorbidities were associated with superior outcomes. Procedural refinements and biomaterial augmentation could further enhance results.

## Introduction

Post-traumatic bone defects and infected non-unions represent some of the most challenging scenarios in orthopedic trauma surgery, particularly in resource-limited settings. Globally, the burden of trauma-related orthopedic injuries is rising significantly, with recent data from the Global Burden of Disease (GBD) 2021 study showing lower limb fractures accounting for a substantial portion of morbidity in low- and middle-income countries (LMICs) [1]. Infected non-unions, characterized by a persistent lack of bone healing in the presence of infection, are especially complex due to the compounding effects of infection, poor vascularization, and biomechanical instability [2]. These cases often require prolonged treatment, multiple surgeries, and may ultimately result in amputation, particularly when treatment options are constrained by limited resources and delayed patient presentation [3].

Traditional strategies for managing such defects, including autografts, allografts, distraction osteogenesis (Ilizarov technique), and even limb amputation, have significant limitations. Autografts are constrained by donor site morbidity and quantity limitations, especially in defects greater than 5 cm [4]. Allografts, while more abundant, carry risks of disease transmission, immunogenicity, and inconsistent integration [5]. Distraction osteogenesis, although effective as shown in studies by Szelerski et al., is highly technique-dependent, prolonged, and has a steep learning curve [6]. Consequently, there is an urgent need for a reliable, reproducible, and biologically favorable method for bone reconstruction [7].

The induced membrane technique (IMT), first introduced by Masquelet, has gained traction in recent decades for the reconstruction of critical-sized bone defects [8]. This two-stage surgical approach involves initial debridement and placement of a polymethylmethacrylate (PMMA) spacer to induce a vascularized membrane, followed by graft placement within the biologically active membrane in a subsequent stage [9]. The membrane secretes growth factors such as vascular endothelial growth factor (VEGF), bone morphogenetic protein-2 (BMP-2), and transforming growth factor-β1 (TGF-β1), which promote angiogenesis and osteogenesis while simultaneously isolating the graft from hostile inflammatory environments [10]. This mechanism is particularly beneficial in infected non-unions, where initial debridement and spacer placement can help control infection and prepare the site for definitive reconstruction [11].

Despite the growing global adoption of IMT, literature on its application in low-resource settings remains limited. Most studies originate from high-income countries where surgical infrastructure, advanced diagnostics, and uninterrupted follow-up care are readily available [5]. In contrast, the implementation of IMT in LMICs like India faces challenges such as delayed presentation, polymicrobial infections, inadequate soft-tissue coverage, and a lack of multidisciplinary support [3]. Nonetheless, the biological advantage offered by the induced membrane makes IMT a compelling option in these scenarios. Preliminary data from African studies suggest favorable outcomes even in challenging environments [3], but more region-specific evidence is required to establish its efficacy and refine treatment protocols.

The current study is situated in a tertiary care hospital in Vadodara, India, where the incidence of high-energy trauma and associated infections is significant. It aims to evaluate the clinical and functional outcomes of IMT in managing infected non-unions and post-traumatic bone defects, with a focus on its feasibility and effectiveness in resource-constrained settings. Specifically, the study investigates union rates, time to union, infection control, and functional outcomes, assessed using standardized scoring systems, building upon previous work by Einhorn and Gerstenfeld on fracture healing mechanisms [12].

Additionally, this study aims to stratify outcomes based on patient age, defect size, etiology (infected vs. aseptic non-unions), and the presence of comorbidities. Stage-wise complications, graft incorporation rates, and return to function are meticulously recorded to provide a comprehensive picture of IMT’s performance in real-world conditions. The results are intended to support clinical decision-making and may contribute to the development of standardized protocols suitable for under-resourced healthcare environments.

By addressing both the biological and logistical complexities of treating large infected bone defects, this study endeavors to validate the IMT as a limb-salvaging, cost-effective, and reproducible alternative to conventional methods. It also opens avenues for future research on biomaterial substitutes for autografts, enhancement of the induced membrane environment, and the role of antibiotic-loaded spacers in infection management.

Against this background, there remains a critical need to evaluate the real-world performance of the IMT in settings where delayed presentation, limited infrastructure, and constrained resources may influence outcomes. While IMT has demonstrated favorable results in controlled and high-resource environments, evidence from LMICs remains limited and heterogeneous. In this context, systematic assessment of both biological success and functional recovery is essential to determine the technique’s reproducibility and clinical value. The present study was therefore designed to assess the clinical and functional outcomes of IMT in the management of infected non-unions and post-traumatic long bone defects in a resource-limited tertiary care setting, with particular attention to union rates, infection control, and patient-related determinants of outcome. The specific objectives of the study are outlined below.

It was hypothesized that the IMT would achieve high rates of bone union and effective infection control, with favorable functional outcomes, in patients with infected non-unions and post-traumatic long bone defects treated in a resource-limited setting.

Objectives

The primary objective of this study was to evaluate the effectiveness of the IMT in the management of infected non-unions and post-traumatic long bone defects in a resource-limited setting, with specific emphasis on radiological union and infection control.

The secondary objectives were to assess functional outcomes using the Association for the Study and Application of the Method of Ilizarov (ASAMI) scoring system and to analyze the influence of patient- and defect-related factors, including age, defect size, etiology (traumatic vs. chronic infected non-union), and associated comorbidities, on treatment outcomes. Additionally, the study aimed to document procedure-related complications, time to union, and overall feasibility of IMT implementation in a constrained healthcare environment.

## Materials and methods

Study design and setting

This was a prospective cohort study conducted between May 2023 and May 2024 at the Department of Orthopedics, Medical College Baroda and Sir Sayajirao General (SSG) Hospital, Vadodara, India. The study was designed to evaluate the clinical and functional outcomes of the two-stage IMT in the management of infected non-unions and post-traumatic long bone defects in resource-limited settings. Ethical clearance was obtained from the Institutional Ethics Committee for Biomedical and Health Research, Medical College Baroda and SSG Hospital (IEC approval no. IECBHR/191-2023), and the study adhered to the ethical standards of the Declaration of Helsinki.

Participants

Participant Selection

Participants were recruited based on well-defined inclusion and exclusion criteria to ensure consistency and clinical relevance.

Sample Size Calculation

The sample size of 35 patients was determined using a confidence level of 95%, expected union rate of 80% based on previous studies [3,5], and a 10% margin of error, yielding a minimum sample of 20 patients.

Inclusion Criteria

Eligible patients were adults aged 18-65 years who presented with infected non-unions of long bones, specifically the femur, tibia, or humerus. The diagnosis of infected non-union was confirmed through a combination of clinical signs, such as sinus tract formation and local inflammation, radiographic evidence of non-union, and positive microbiological cultures. Only patients with segmental bone loss greater than 3 cm, primarily resulting from post-traumatic osteomyelitis or delayed union, were considered for inclusion. Furthermore, all participants were required to be mentally competent and willing to undergo the staged surgical protocol of the IMT, providing written informed consent before enrollment.

Exclusion Criteria

Patients were excluded from the study if they exhibited active systemic infection or sepsis at the time of screening. Additional exclusion criteria included immunocompromised states (such as HIV/AIDS or ongoing chemotherapy), inability to comply with postoperative follow-up protocols, and inadequate soft-tissue coverage necessitating plastic surgical reconstruction services that were not available at the study center. Pregnant and lactating women were also excluded to avoid potential surgical and anesthetic risks.

Study parameters

The primary outcome parameters included union rate, time to union, infection control rate, and functional recovery, assessed using the ASAMI scoring system.

Following the application of these criteria, a total of 30 patients meeting all eligibility requirements were enrolled consecutively over the study period for prospective evaluation.

Preoperative evaluation

Each patient underwent a thorough clinical evaluation, including pain assessment, range of motion measurement, limb-length discrepancy documentation, and evaluation of local signs of infection. Laboratory investigations included complete blood count, erythrocyte sedimentation rate (ESR), C-reactive protein (CRP), and renal and liver function tests. Radiographs (anteroposterior and lateral) of the affected limb were obtained to evaluate the extent of non-union and bone loss. Magnetic resonance imaging (MRI) and/or computed tomography (CT) scans were performed in selected cases to assess marrow involvement and plan surgical debridement.

Surgical protocol: two-stage IMT

The surgical management of infected non-unions in this study followed the two-stage IMT initially described by Masquelet, with practical adaptations to suit a resource-limited healthcare setting. This standardized protocol was employed to address both infection control and bone defect reconstruction.

First Stage: Debridement and Spacer Insertion

The first stage involved thorough debridement and placement of an antibiotic-loaded PMMA cement spacer. Under spinal or general anesthesia, a wide surgical exposure of the non-union site was achieved. Radical debridement was carried out meticulously, removing all necrotic bone, fibrous tissue, and sequestra until healthy, bleeding bone was visualized, a hallmark of adequate debridement known as the “paprika sign.” Intraoperative deep tissue samples were obtained for aerobic and anaerobic cultures to guide targeted antibiotic therapy. Intramedullary canals were reamed and thoroughly irrigated to clear residual infection.

Following debridement, the size of the bone defect was measured, and a PMMA spacer impregnated with antibiotics was molded to conform to the defect morphology. Postoperatively, all patients were initiated on empirical broad-spectrum intravenous antibiotic therapy immediately following radical debridement. The initial regimen typically consisted of intravenous vancomycin (1 g twice daily) combined with a third-generation cephalosporin or piperacillin-tazobactam, according to institutional protocol and patient renal function. Intravenous antibiotics were administered for a minimum duration of four weeks and extended up to six weeks in cases of polymicrobial infection, delayed wound healing, persistently elevated inflammatory markers, or ongoing clinical suspicion of infection. Following completion of intravenous therapy, selected patients received oral antibiotics for an additional two to four weeks, where clinically indicated. Antibiotic therapy was discontinued only when clinical signs of infection had resolved and inflammatory markers (ESR and CRP) had normalized on at least two consecutive assessments performed two weeks apart. For skeletal stabilization, internal fixation using locking compression plates or intramedullary nails was used in 24 cases, while external fixation was reserved for six patients who had compromised soft tissue envelopes or a higher risk of infection recurrence. In all cases, primary wound closure was achieved without tension.

Postoperatively, patients were started on culture-specific intravenous antibiotics for a duration of four to six weeks, tailored based on microbial sensitivity. This was followed by an oral antibiotic regimen, when clinically indicated, ensuring continued suppression of residual infection.

Second Stage: Spacer Removal and Bone Grafting

The second stage was scheduled between six and eight weeks after the initial procedure, contingent upon satisfactory clinical and laboratory evidence of infection resolution. Criteria for proceeding included normalization of inflammatory markers such as ESR and CRP, along with the absence of local signs of infection. During this stage, the previously induced membrane was carefully opened, and the PMMA spacer was removed while preserving the vascularized membrane envelope.

The resulting bone defect was then filled with autologous cancellous bone graft harvested from the ipsilateral iliac crest. In patients with larger defects exceeding 5 cm, bone grafts were supplemented with allografts or osteoconductive materials such as hydroxyapatite granules to augment graft volume. Mechanical stabilization was re-evaluated, and internal fixation was either maintained or revised as necessary to ensure optimal stability and facilitate bone healing.

Soft tissue closure was performed meticulously without excessive tension, and closed-suction drains were placed to prevent postoperative hematoma formation. Postoperative rehabilitation included early mobilization, with patients encouraged to initiate partial weight-bearing as tolerated starting from the second postoperative day, supporting functional recovery while minimizing stress at the graft site.

Outcome measures

Patients were prospectively followed up at six weeks, three months, six months, and 12 months postoperatively to evaluate clinical, radiological, and functional outcomes. Radiological union was assessed through standard anteroposterior and lateral radiographs, with bone healing defined as the presence of bridging callus across at least three out of four cortices. Infection control was closely monitored throughout the follow-up period, with reinfection indicated by the recurrence of draining sinus tracts, persistently elevated inflammatory markers (ESR/CRP), or positive culture reports. Successful eradication of infection was defined as the complete absence of clinical and laboratory evidence of infection for a minimum of six months following bone grafting. Functional recovery was evaluated using the ASAMI scoring system, which assesses both bone and functional outcomes. Bone results were graded as excellent (achieving union with no infection, deformity <7°, and limb-length discrepancy <2.5 cm), good (union with one of the aforementioned issues), fair (union with two or more issues), and poor (persistent non-union or infection). Functional results were categorized based on parameters including pain, gait (limp), joint stiffness, and the patient’s ability to return to pre-injury occupational or daily activities, thereby providing a comprehensive measure of overall patient recovery and quality of life.

Statistical analysis

Data were entered into Microsoft Excel and analyzed using IBM SPSS Statistics, version 25.0 (IBM Corp., Armonk, NY). Continuous variables (e.g., age and defect size) were expressed as mean ± standard deviation. Categorical data (e.g., ASAMI scores) were expressed as frequencies and percentages. The association between defect size and union rate was tested using chi-square or Fisher’s exact test as appropriate. A p-value of <0.05 was considered statistically significant. Due to the small sample size, multivariate analysis was not feasible, and findings from stratified analyses should be interpreted cautiously.

## Results

This prospective observational cohort study was conducted from May 2023 to May 2024 at the Department of Orthopedics, Medical College Baroda and SSG Hospital. A total of 35 patients with infected non-union or post-traumatic long bone fractures associated with bone loss were initially evaluated. Of these, 10 patients were excluded as they did not meet the inclusion criteria, and five patients were lost to follow-up. Thus, the final analysis comprised 20 patients who were treated using the IMT.

The summary of patient demographics and clinical profiles of study participants is summarized in Table 1. The age distribution showed that 13 patients (65%) were under the age of 40 years, while the remaining seven patients (35%) were between 40 and 60 years. There was a marked male predominance in the study population, with 15 male patients (75%) and five female patients (25%). In terms of occupation, 12 patients (60%) were laborers, five patients (25%) were office workers, and three patients (15%) were housewives. This indicates a higher incidence of such injuries in individuals involved in physically demanding or high-risk occupational settings. 

**Table 1:** Demographic and clinical profile

Category/group	Frequency (%)
Age group	
<40 years	13 (65%)
40-60 years	7 (35%)
Gender	
Male	15 (75%)
Female	5 (25%)
Occupation	
Laborer	12 (60%)
Office worker	5 (25%)
Housewife	3 (15%)
Etiology	
Trauma	15 (75%)
Infected non-union	5 (25%)

The surgical and clinical characteristics, including fixation method and laterality, are presented in Table 2. Trauma was the most common etiological factor, observed in 15 patients (75%), whereas five patients (25%) had chronic infected non-unions. The most commonly used fixation method was plate fixation, employed in 16 patients (80%), while intramedullary nailing was performed in four patients (20%). Laterality showed right-sided involvement in 11 cases (55%) and left-sided involvement in nine cases (45%).

**Table 2:** Surgical and clinical characteristics of patients

Category/group	Frequency (%)
Fixation method	
Plate	16 (80%)
Nail	4 (20%)
Laterality	
Right	11 (55%)
Left	9 (45%)
Bone defect size	
<5 cm	8 (40%)
5-10 cm	10 (50%)
>10 cm	2 (10%)
Local condition	
Open fracture	15 (75%)
Local infection	5 (25%)

The length of the bone defect varied among patients. Eight patients (40%) had defects less than 5 cm, 10 patients (50%) had defects between 5 and 10 cm, and only two patients (10%) had defects greater than 10 cm. A large proportion of patients (15 out of 20; 75%) presented with open wound fractures, while the remaining five patients (25%) had localized bone infections. The distribution of affected bones among the study population is presented in Table 3. The femur was the most commonly involved bone, accounting for 10 cases (50%), followed by the tibia in eight patients (40%). Upper limb involvement was comparatively less frequent, with one case each involving the humerus (5%) and ulna (5%).

**Table 3:** Bone-wise distribution in patients

Type of bone	Frequency (%)
Femur	10 (50)
Tibia	8 (40)
Humerus	1 (5)
Ulna	1 (5)

Table 4 summarizes co-morbidities in patients. Regarding systemic comorbidities, two patients (10%) were known diabetics, and one patient (5%) had hypertension, while 17 patients (85%) had no associated medical illness. 

**Table 4:** Comorbidity profile of the patients

Comorbidity	Frequency (%)
Diabetes mellitus	2 (10%)
Hypertension	1 (5%)
None	17 (85%)

The outcomes were assessed using the ASAMI scoring system and are shown in Table 5. An excellent outcome was observed in five patients (25%), a good outcome in 11 patients (55%), a fair outcome in three patients (15%), and a poor outcome in one patient (5%). Therefore, 80% of patients achieved a satisfactory result (excellent or good), supporting the effectiveness of the IMT in this population.

**Table 5:** Outcomes and complications in patients ASAMI, Association for the Study and Application of the Method of Ilizarov

Category/group	Frequency (%)
ASAMI outcome	
Excellent	5 (25%)
Good	11 (55%)
Fair	3 (15%)
Poor	1 (5%)
Complication	
Deformity	2 (10%)
Infection	1 (5%)
None	17 (85%)

Stratified analysis showed that younger patients tended to have better outcomes, as shown in Table 6. Among the 13 patients aged 20-40 years (65%), five (25%) had excellent outcomes, six (30%) had good outcomes, and two (10%) had poor outcomes. Among the seven patients aged 40-60 years (35%), one (5%) had an excellent result, four (20%) had good results, one (5%) had a fair result, and one (5%) had a poor result. This distribution suggests a higher rate of bone regeneration and healing in younger individuals compared to older patients.

**Table 6:** Combined stratified outcome analysis of patients

Stratification	Excellent	Good	Fair	Poor
Gender				
Male (n = 15)	4 (20%)	9 (45%)	0 (0%)	2 (10%)
Female (n = 5)	1 (5%)	2 (10%)	1 (5%)	1 (5%)
Occupation				
Laborer (n = 12)	3 (15%)	7 (35%)	0 (0%)	2 (10%)
Office worker (n = 5)	1 (5%)	3 (15%)	0 (0%)	1 (5%)
Housewife (n = 3)	1 (5%)	1 (5%)	1 (5%)	0 (0%)
Etiology				
Trauma (n = 15)	4 (20%)	8 (40%)	1 (5%)	2 (10%)
Infected non-union (n = 5)	1 (5%)	3 (15%)	0 (0%)	1 (5%)
Bone defect				
Size: defect <5 cm (n = 8)	3 (15%)	4 (20%)	0 (0%)	1 (5%)
Bone defect size: defect 5-10 cm (n = 10)	2 (10%)	6 (30%)	1 (5%)	1 (5%)
Bone defect size: defect >10 cm (n = 2)	0 (0%)	1 (5%)	0 (0%)	1 (5%)
Comorbidity				
Diabetic (n = 2)	0 (0%)	2 (10%)	0 (0%)	0 (0%)
Hypertensive (n = 1)	1 (5%)	0 (0%)	0 (0%)	0 (0%)
No comorbidity (n = 17)	4 (20%)	9 (45%)	1 (5%)	3 (15%)

Gender-based outcome analysis revealed that of the 15 male patients (75%), four (20%) achieved excellent outcomes, nine (45%) had good outcomes, and two (10%) had poor outcomes. Among the five female patients (25%), one (5%) had an excellent result, two (10%) had good results, one (5%) had a fair outcome, and one (5%) had a poor result. While outcomes were similar across genders, males had slightly better results, possibly due to their higher representation and more robust bone biology.

Occupationally, laborers (n = 12; 60%) demonstrated relatively favorable outcomes, with three (15%) achieving excellent results, seven (35%) having good outcomes, and two (10%) experiencing poor results. Among office workers (n = 5; 25%), one (5%) had an excellent result, three (15%) had good results, and one (5%) had a poor outcome. Of the three housewives (15%), one (5%) each had excellent, good, and fair results, respectively. These findings may be influenced by the predominance of trauma in laborers and relatively earlier access to care.

When outcomes were analyzed according to etiology, patients with trauma (n = 15; 75%) had better results, with four (20%) excellent, eight (40%) good, one (5%) fair, and two (10%) poor outcomes. In contrast, among the five patients (25%) with infected non-union, one (5%) had an excellent result, three (15%) had good results, and one (5%) had a poor outcome. This suggests that traumatic bone defects, when promptly managed, may have better outcomes compared to chronic infected non-unions.

Bone defect size was another important predictor of treatment outcome. Among the eight patients (40%) with bone defects <5 cm, three (15%) had excellent outcomes, four (20%) had good outcomes, and one (5%) had a poor result. Among the 10 patients (50%) with 5-10 cm defects, two (10%) had excellent outcomes, six (30%) had good outcomes, one (5%) had a fair result, and one (5%) had a poor result. In contrast, of the two patients (10%) with defects >10 cm, one (5%) had a good outcome and one (5%) had a poor result. Thus, patients with smaller defects (<10 cm) responded more favorably to IMT.

Comorbidities also influenced outcomes. Among the two diabetic patients (10%), both had good outcomes. The hypertensive patient (5%) had an excellent result. Among the 17 patients (85%) without any comorbid illness, four (20%) had excellent outcomes, nine (45%) had good outcomes, one (5%) had a fair result, and three (15%) had poor outcomes. Although comorbidity was not universally associated with poor outcomes, patients without systemic disease tended to have more favorable results.

Postoperative complications were encountered in three patients (15%). Two patients developed deformity due to malalignment during bone reconstruction, and one patient had a persistent infection at the surgical site that required long-term antibiotics and debridement. All three of these patients had poor outcomes. The remaining 17 patients (85%) did not experience any major complications and mostly had excellent or good results, highlighting the importance of preventing complications to optimize outcomes.

## Discussion

The present study evaluated the clinical and functional outcomes of the IMT for managing infected non-unions and post-traumatic bone defects in a resource-constrained tertiary care center in India. Our findings affirm that IMT achieved satisfactory outcomes, defined as excellent or good ASAMI scores, in 80% of patients, underscoring its reliability in low-resource environments where advanced limb reconstruction options are often limited. These findings are particularly relevant to LMICs, where the burden of trauma-related orthopedic injuries continues to rise, as demonstrated by the GBD 2021 study showing a disproportionate incidence of lower limb fractures in such regions [1].

The success rate observed in our cohort is comparable to the existing literature from both high- and low-resource settings. Kombate et al. reported a 73% success rate using IMT in a West African context [3], while Giannoudis et al. documented 75-85% union rates in high-income countries with superior surgical infrastructure and early access to treatment [5]. Tudor et al. described an even higher success rate of 90% in a European center, likely attributable to enhanced perioperative care, availability of bone substitutes, and early-stage intervention [8]. In contrast, LMICs often encounter delayed presentations, limited soft-tissue coverage, and inadequate diagnostic support, factors that may account for the slight disparity in outcomes. Our findings echo Wang et al., who highlighted the negative impact of systemic barriers, such as poor access to timely imaging and definitive surgical care, on musculoskeletal trauma outcomes in LMICs [1].

Age emerged as a significant prognostic factor in our study, consistent with prior evidence. Patients under 40 years of age demonstrated an 85% success rate, as opposed to 71% in older individuals. This supports the biological principle of age-related osteogenic potential, extensively described by Einhorn and Gerstenfeld, who noted superior angiogenesis and mesenchymal stem cell activity in younger patients [12]. Bone defect size also played a crucial role in outcomes: patients with defects smaller than 5 cm experienced an 88% success rate, whereas those with defects larger than 10 cm achieved only a 50% success rate. These findings corroborate Piacentini et al.’s assertion of a “critical defect size” threshold beyond which autograft alone becomes insufficient for optimal regeneration [4].

In addition to demographic variables, this study explored the impact of comorbidities and socioeconomic factors. Male patients exhibited marginally better outcomes than females (80% vs. 60%), possibly due to sex-based differences in bone healing capacity and occupational exposure to early mobility and loading forces. Laborers, who comprised 60% of the study population, tended to seek care earlier and demonstrated high motivation for functional recovery, both of which may have contributed to improved outcomes. Furthermore, the presence of systemic comorbidities such as diabetes and hypertension adversely affected outcomes; patients without comorbidities had an 82% success rate compared to 66% in those with chronic illnesses. These observations are consistent with the findings of Grgurević et al., who emphasized the role of systemic lipid metabolism dysregulation and oxidative stress in impairing bone regeneration and contributing to non-union formation [13].

The infection control protocol in this study, consisting of radical debridement, vancomycin- and gentamicin-loaded PMMA spacers, and culture-specific systemic antibiotics, proved highly effective, achieving a reinfection rate of just 5%. This result is aligned with prior studies by Chloros et al. and Liu et al., who underscored the importance of antibiotic-impregnated spacers in reducing bacterial biofilm persistence and enhancing the inflammatory milieu for membrane formation [9,14]. Furthermore, the visual confirmation of viable bleeding bone, known as the “paprika sign,” remains a critical intraoperative marker of debridement adequacy and has been emphasized in foundational studies on IMT-induced granulation by Wang et al. [10].

Despite the favorable outcomes, several complications were documented, including deformity, persistent infection, and delayed union. Approximately 15% of patients experienced these adverse events, all of whom scored poorly on the ASAMI scale. These results reiterate the importance of meticulous intraoperative technique, particularly in alignment, fixation stability, and soft tissue handling. The need for intraoperative imaging and plastic surgical support, often unavailable in LMIC hospitals, presents a tangible limitation in replicating high-income country protocols in these settings [15].

The study’s limitations include its relatively small sample size (n = 20), which restricts statistical power and precludes multivariate analysis. Moreover, the 12-month follow-up period, while sufficient for preliminary assessment, may not be adequate to capture long-term complications such as graft resorption, recurrent infection, or reoperation rates. As Calori et al. have pointed out, the biological integration of large grafts often continues beyond one year, and late complications are not uncommon [16]. Another constraint was the limited access to advanced imaging, such as CT volumetry or MRI, which might have provided more objective parameters for evaluating graft incorporation and union.

Nonetheless, the clinical implications of our findings are significant, particularly for settings where cost, access, and surgical training pose formidable barriers. IMT remains a biologically favorable and cost-effective technique, utilizing autologous graft material and affordable fixation methods that are widely available in LMICs [3]. Our study protocol, emphasizing staged debridement, adequate infection control, and a second-stage grafting within a mature membrane, closely mirrors the key procedural tenets outlined by Giannoudis et al., reinforcing its reproducibility even in modest clinical environments [11].

Looking ahead, several research directions are warranted to further improve outcomes. For large defects exceeding 10 cm or those in patients with limited graft availability, biomaterial augmentation using hydroxyapatite, β-tricalcium phosphate (β-TCP), or collagen composites may provide synergistic scaffolding support. Calori et al. have demonstrated promising outcomes using synthetic bone substitutes in combination with autograft, particularly in complex reconstructions [16]. Additionally, telemedicine platforms can be leveraged to improve patient compliance and follow-up adherence in rural areas, as evidenced by Kumar et al., who demonstrated the feasibility and acceptability of virtual consultations in orthopedic populations [17]. Finally, emerging research into systemic modulators of bone healing, including lipid-lowering agents and antioxidants, may open novel therapeutic avenues to enhance regeneration in metabolically compromised patients [13].

This study has important limitations that should be considered when interpreting the findings. The relatively small final sample size limited statistical power and precluded multivariate modeling to adjust for potential confounders. Although a standardized two-stage IMT protocol was followed, minor variations in fixation strategy, graft supplementation for larger defects, and individualized antibiotic duration may have introduced treatment heterogeneity. Furthermore, while the ASAMI scoring system is widely accepted, it incorporates clinical judgment and may be subject to interobserver variability. Stratified subgroup analyses were largely descriptive due to sample size constraints, and inferential comparisons should therefore be interpreted cautiously. The 12-month follow-up period may not fully capture late complications such as graft remodeling failure or delayed recurrence of infection. Finally, the exclusion of patients lost to follow-up may have introduced attrition bias. Larger multicenter studies with longer follow-up and standardized treatment pathways are needed to validate and expand upon these findings.

## Conclusions

This prospective cohort study suggests that the IMT is a practical and adaptable option for managing complex post-traumatic bone defects and infected non-unions in a resource-limited setting. Satisfactory outcomes were achieved in 80% of patients, with favorable trends observed among younger individuals, those with smaller defects, and patients without systemic comorbidities. Infection control was maintained in the majority of cases, and functional recovery was generally encouraging at 12 months. However, these findings should be interpreted in light of the study’s small sample size, single-center design, and absence of a control group. While IMT appears to be a feasible limb-salvage strategy in low- and middle-income settings, larger multicenter studies with longer follow-up and comparative designs are required to confirm its effectiveness and define its role relative to alternative reconstructive techniques.
